# Quantum Chemical Simulation of the *Q*_y_ Absorption Spectrum of Zn Chlorin Aggregates for Artificial Photosynthesis

**DOI:** 10.3390/molecules26041086

**Published:** 2021-02-19

**Authors:** Zhimo Wang, Bingbing Suo, Shiwei Yin, Wenli Zou

**Affiliations:** 1 Institute of Modern Physics, Northwest University, and Shaanxi Key Laboratory for Theoretical Physics Frontiers, Xi’an 710127, China; wangzhimo@stumail.nwu.edu.cn; 2 Key Laboratory for Macromolecular Science of Shaanxi Province, School of Chemistry and Chemical Engineering, Shaanxi Normal University, Xi’an 710062, China; yin_sw@snnu.edu.cn

**Keywords:** *Q_y_* absorption spectra, explicit solvent model, layered aggregates, förster coupling theory, time-dependent density functional theory

## Abstract

Zn chlorin (Znchl) is easy to synthesize and has similar optical properties to those of bacteriochlorophyll c in the nature, which is expected to be used as a light-harvesting antenna system in artificial photosynthesis. In order to further explore the optical characteristics of Znchl, various sizes of a parallel layered Znchl-aggregate model and the THF-Znchl explicit solvent monomer model were constructed in this study, and their $Q_{y}$ excited state properties were simulated by using time-dependent density functional theory (TDDFT) and exciton theory. For the Znchl monomer, with a combination of the explicit solvent model and the implicit solvation model based on density (SMD), the calculated $Q_{y}$ excitation energy agreed very well with the experimental one. The Znchl aggregates may be simplified to a Zn36 model to reproduce the experimental $Q_{y}$ absorption spectrum by the Förster coupling theory. The proposed Znchl aggregate model provides a good foundation for the future exploration of other properties of Znchl and simulations of artificial light-harvesting antennas. The results also indicate that J-aggregrates along z-direction, due to intermolecular coordination bonds, are the dominant factor in extending the $Q_{y}$ band of Znchl into the near infrared region.

## 1. Introduction

The chlorosomes in green photosynthetic bacteria are the largest light-harvesting antennae in the nature, which are composed of more than 200,000 bacteriochlorophyll (Bchl) c, d, and e molecules [1]. They can collect solar energy in low-light conditions, and the acquired energy may be transferred to the photosynthetic reaction center very quickly, so green photosynthetic bacteria can be found in conditions of low light [2]. Compared with other light-harvesting antenna systems [3], the chlorosomes do not need complex proteins to provide a scaffold in the formation process, and therefore their light-harvesting capacity is more efficient [4]. Due to the above advantages, chlorosomes are widely used as a model of artificial light-harvesting antenna [5,6].

Due to large volumes (from 98 × 38 × 11 to 209 × 104 × 29 nm) and heterogeneous composition pigments [7], the structures of chlorosomes have not been adequately identified at the atomic level. Several teams simulated and characterized the structures of chlorosomes in natural green sulfur bacteria by means of cryo-electron microscopy, and proposed three possible structures, i.e., layered [8], tubular [9], and spiral [10] ones, respectively, which provide some clues for the construction of artificial light capture antenna models.

**Figure 1:**
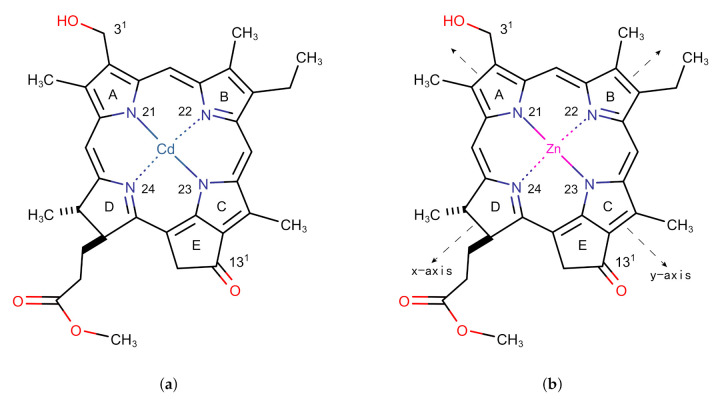
Monomer models of (**a**) Cd chlorin and (**b**) Zn chlorin.

**Figure 2:**
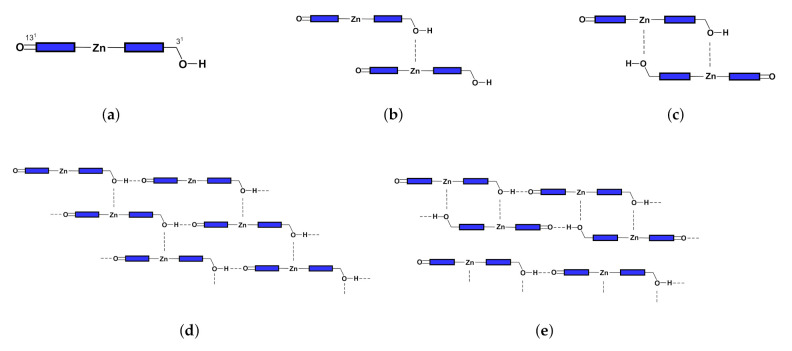
Configurations of (**a**) monomer, (**b**) open dimer, (**c**) closed dimer, (**d**) open aggregate, and (**e**) close aggregate.

Inspired by the early studies of chlorosomes, Tamiak et al. [11] synthesized Zn-centered chlorin (Zn chlorin, or Znchl for short) by replacing the central Mg atom in chlorophyll-a [12,13] with a Zn atom to simulate Bchl c, the dominant component of chlorosomes in the nature. Znchl (see Figure 1b) and Bchl c molecules have similar optical properties and can self-assemble into aggregate structures in non-polar solvents, but the former is much easier to synthesize. Znchl exists as a monomer in pure THF but self-assembles into supramolecular aggregates in 1% (vol/vol) THF–hexane [14]. Infra-red spectra indicated that $3^{1}-OH$ in the Znchl aggregates could form an O–H intermolecular hydrogen bond to $13^{1}-O$ of an adjacent molecule and a coordination bond to Zn in the other Znchl center [15]. Therefore, two possible stepped layered aggregate structures, i.e., the parallel stack model (see Figure 2d) and the reverse parallel stack model (Figure 2e), may be proposed, which are suitable structural models for the study of the artificial light-harvesting antenna systems. Interestingly, the $Q_{y}$ absorption spectrum of Znchl aggregates (747 nm) is red-shifted by 1900 cm${}^{-1}$ relative to that of the Znchl monomer [14], meaning that the former captures light easier, and can extend the absorption spectrum of the monomer into the near infrared region. The infrared wavelength region contains a lot of energy and makes up a large percentage of sunlight [2], which is a good source of photons, which drive photosynthesis. Therefore, it is worth paying attention to the causes of red shift in the absorption spectrum. So far, some teams have studied the structures and the characteristics of chlorosomes in their excited states [16,17], which are helpful for the study of the influential factors in these artificial synthesis models. To the best of our knowledge, however, no theoretical studies on the Znchl monomer and its aggregates have been completed until now.

In this study, a Znchl monomer model was theoretically constructed at first. It was subsequently used to study the possible parallel-stacked, stepped, layered aggregates. The absorption spectra of the Znchl monomer and its aggregates were simulated by time-dependent density functional theory (TDDFT) [18,19] combined with classical exciton theory [20,21,22], and the effects of intermolecular coupling and solvent environment on the absorption spectra of isolated molecules were investigated as well. We also explored the key factors for making the $Q_{y}$ band of the Znchl monomer remarkably red-shifted, allowing it to effectively harvest the near infrared sunlight. In addition, the theoretical model and the related Hamiltonian provide an indispensable basis for the further study of ultra-fast spectra and energy transfer of the artificial light-harvesting antenna.

## 2. Computational Methods

### 2.1. The Structure and $Q_{y}$ Excitation Energy of the Znchl Monomer

The structure of the Znchl monomer was derived from the Cd chlorin molecular model in the CCDC crystal database (identifier 224617; see Figure 1a) [23]; the Cd atom in the latter was replaced by Zn (Figure 1b). In order to simulate the solvent environment of the Znchl monomer in experiments [14], both explicit and implicit solution models have been implemented. The monomer in the explicit solvent model is the fifth axial ligand, THF, with an additional Zn atom in the center of the optimized monomer model. As in the chlorophyll ligands in nature [17], an axial coordination between the THF solvent molecule and the Zn atom is formed in Znchl with the initial bond distance of 2.169 Å between the O in THF and Zn. The structure of the Znchl monomer at the ground state was optimized at the B3LYP [24] level of theory with the 6-31G(d) basis set [25,26] in gas phase, whereas the excited states were calculated either in gas phase or in implicit THF solution by TDDFT with three range-separated functionals, LC-ωPBE [27], ωB97X [28], and CAM-B3LYP [29], combined with the def2-TZVP basis set [30]. For the complex model of a monomer in explicit solvent, the structure at the ground state was optimized by B3LYP/6-31G(d), with and without Grimme’s D3 dispersion correction [31], and the excited states were calculated using the same methods as done in the above TDDFT calculations of the Znchl monomer.

### 2.2. Exciton Hamiltonian

Usually, the capture and transfer of light energy are related mainly to the first excited state of the light capture antenna, and therefore only the $Q_{y}$ excited state of Znchl aggregate is concerned in this work. Due to no complex proteins and other substances in the Znchl aggregate, the classical Hamiltonian [32] may be written as

(1) $$\hat{H}=\sum_{m=1}^{N}\varepsilon_{m}{\hat{B}}_{m}^{+}{\hat{B}}_{m}+\frac{1}{2}\sum_{{}_{m,l=1}^{m\neq l}}^{N}H_{ml}\left({\hat{B}}_{l}^{+}{\hat{B}}_{m}+{\hat{B}}_{m}^{+}{\hat{B}}_{l}\right),$$

with $\varepsilon_{m}=E_{m}+\delta m$, where $E_{m}$ is the electronic excitation energy of the *m*-th single molecule, δm is the shift induced by the surrounding solvent matrix, $H_{ml}$ is the coupling strength between the *m*-th and *l*-th molecules, and ${\hat{B}}_{m}^{+}$ and ${\hat{B}}_{m}$ are respectively Pauli creation and annihilation operators of exciton for the *m*-th molecule.

Let the ground state wave function of the *m*-th molecule be $\phi_{g_{m}}$; then the ground state wave function of the aggregate system may be expressed as

(2) $$\Phi_{g}=\phi_{g_{1}}\phi_{g_{2}}...\phi_{g_{N}}=\prod_{m=1}^{N}\phi_{g_{m}}.$$

If the *m*-th molecule is in the first excited state, the corresponding wave function of the aggregate system is

(3) $$\Phi_{e_{m}}=\phi_{g_{1}}\phi_{g_{2}}...\phi_{e_{m}}...\phi_{g_{N}}={\hat{B}}_{m}^{+}\Phi_{g}.$$

Assuming that there is no overlap between the molecular wave functions, the excited state wave function of the aggregate system in the zero-order approximation has the form

(4) $$\Psi_{e}=\sum_{m=1}^{N}C_{e_{m}}\Phi_{e_{m}}=\sum_{m=1}^{N}C_{e_{m}}{\hat{B}}_{m}^{+}\Phi_{g}.$$

Using Equations (1) and (3), the Hamiltonian matrix of different aggregates may be constructed,

(5) $$H_{AGG}=\left[\begin{array}{ccccc} H_{11} & ... & H_{1m} & ... & H_{1N}\\ ... & ... & ... & ... & ...\\ H_{N1} & ... & H_{Nm} & ... & H_{NN} \end{array}\right]=\left[\begin{array}{ccccc} \epsilon_{1} & ... & H_{1m} & ... & H_{1N}\\ ... & ... & ... & ... & ...\\ H_{N1} & ... & H_{Nm} & ... & \epsilon_{N} \end{array}\right],$$

where the diagonal element $\epsilon_{N}$ is the site energy of the *N*-th molecule, and the off-diagonal element $H_{Nm}$ is the coupling strength between the *N*-th and *m*-th molecules.

### 2.3. Coupling Strength

With the help of the point dipole approximation and the solvent shielding effect, the coupling strength $H_{ml}$ in Equation (1) may be calculated by the Förster coupling theory [33] according to

(6) $$H_{ml}=\frac{f}{4\pi \varepsilon_{0}}\left[\frac{{\hat{\mu }}_{m}\cdot {\hat{\mu }}_{l}}{R_{ml}^{3}}-\frac{3\left({\hat{\mu }}_{m}\cdot {\hat{R}}_{ml}\right)\left({\hat{\mu }}_{l}\cdot {\hat{R}}_{ml}\right)}{R_{ml}^{5}}\right],$$

where $\varepsilon_{0}$ is the vacuum dielectric constant, ${\hat{\mu }}_{m}$ is the transition dipole moment vector operator for the *m*-th molecule, ${\hat{R}}_{ml}$ is the distance vector between two molecules, and *f* is the solvent shielding factor related to $R_{ml}$

(7) $$f\left(R_{ml}\right)=A\exp\left(-\beta R_{ml}+f_{0}\right).$$

Since the dielectric constant of the 1% (vol/vol) THF–hexane solution (ϵ = 1.89$\epsilon_{0}$) is close to 2$\epsilon_{0}$, the factors A, β, and $f_{0}$ in Equation (7) were estimated to be 2.68, 0.27, and 0.54, respectively, according to reference [34].

It should be pointed out that there are many more accurate methods [35,36,37,38,39] than the point dipole approximation, such as the transition density cube [35,36] method, the configuration interaction exciton [37] method, and others [38,39]. We finally choose the point dipole approximation because of the following considerations. First of all, we aimed to explore the effect of aggregate size on the molecular absorption spectrum. Due to the large and diverse aggregate structure, it would not be practical to use other methods to calculate intermolecular coupling. Second, previous studies have shown that exciton theory combined with a dipole approximation correctly describes the absorption spectra of chlorosome-like [40,41] and other light-capture antennas [42,43].

The topological analysis of electron density distribution was also performed by the quantum theory of atoms in molecules (QTAIM) [44] at (3,-1) bond critical points (BCPs) to investigate the interactions between the two fragments in a Znchl dimer stacked in z-direction (Zn-zdimer). The Multiwfn program [45] was used for the QTAIM analysis.

### 2.4. Structure and $Q_{y}$ Absorption Spectrum of Aggregate

To obtain the aggregate structure, a monomer may be expanded in any one of three directions *x*, *y*, and *z*, where *y* and *x* are the N21–N23 and N22–N24 (see Figure 1 for the numbering) bond directions, respectively, and *z* is perpendicular to the chlorin ring. In our simulation, an re-optimized Znchl monomer was repeated 24 times in each direction (denoted as Zn24-X/Y/Z), and the coupling strength between the first molecule and the remaining 23 molecules in the Zn24-X/Y/Z aggregate ($H_{1j}$) was calculated by the Förster coupling theory. Finally, the constructed largest aggregate adoped the size of $L_{jx}\times L_{jy}\times L_{jz}$, where jx/jy/jz may be estimated by a small enough $H_{1j}$ value (see below) in the three directions.

The structure of the aggregate was optimized by the L-BFGS [46] algorithm at the PBE [47]/DZVP-MOLOPT-SR-GTH [48] level of theory, implemented in the CP2K package [49,50,51]. In addition, the first excited state of an individual molecule in the aggregate and its transition electric dipole moment were also calculated by TDDFT with the ωB97X hybrid functional [28] using the Gaussian 09 program package [52]. Since the transition electric dipole moment is greatly influenced by diffuse basis functions and surrounding environments, the def2-SVPD [53] basis set and the implicit solvation model based on density (SMD) [54] with 1% (vol/vol) THF–hexane were employed.

For the $Q_{y}$ absorption spectrum of the aggregate models, the Gaussian linear broadening function was used to simulate the inhomogeneous broadening of the spectrum. After the Hamiltonian matrix of the aggregate in Equation (5) was diagonalized, the absorption intensity at a given wavelength ν was computed by

(8) $$A\left(\nu \right)=\frac{\nu }{N\sigma \sqrt{2\pi }}\sum_{i=1}^{N}M_{i}^{2}exp\left[-\frac{{\left(h\nu -E_{i}\right)}^{2}}{2\sigma^{2}}\right],$$

where σ is the standard variance of the Gaussian distribution, 0.0248 here, and $E_{i}$ and $M_{i}$ are respectively the excitation energy and transition dipole moment of the *i*th eigenvector.

## 3. Results and Discussion

### 3.1. The Znchl Monomer and THF-Znchl

**Figure 3:**
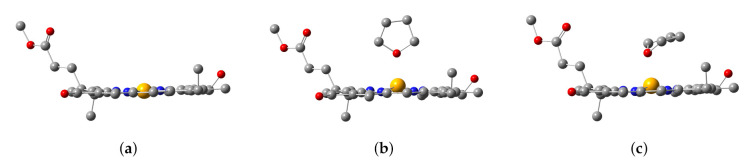
Configurations of (**a**) Znchl monomer (model 1), (**b**) THF-Znchl without dispersion correction (model 2), and (**c**) THF-Znchl with dispersion correction (model 3).

After geometric optimizations of the Znchl monomer models by B3LYP/6-31G(d) either in gas phase or in the explicit solvent model, three structures were obtained, i.e., the Znchl monomer (model 1), and THF-Znchl models without (model 2) and with Grimme’s D3 dispersion correction (model 3), and their side views are shown in Figure 3. It can be seen that the Zn atom is almost in the chlorin ring plane in model 1, but leaves the plane in model 2 and model 3 due to the effect of THF axial ligand; the dispersion correction has a great influence on the configurations of the latter two complexes. The key structural parameters of the three models have been collected in Table 1. Since the Zn–N21 and Zn–N23 bond lengths are very similar, they were selected to define the departure of Zn from the chlorin plane, which may be estimated approximately by the following formula of DOOP (out-of-plane distance).

(9) $$DOOP=\frac{\left(R_{Zn-N21}+R_{Zn-N23}\right)}{2}\cos\left(\frac{{∠}_{N21-Zn-N23}}{2}\right).$$

As seen in Table 1, the DOOP value increases significantly after the coordination of THF with Znchl.

Three typical range separation functionals LC-ωPBE, ωB97X, and CAM-B3LYP combined with the def2-TZVP basis set were used to calculate the first excited states of the three models in THF solution through the implicit solvent model SMD, and the excitation energies and oscillator strengths (*f*) are given in Table 2. As the short-range exact-exchange components in these functionals increased (0.0%, 15.77%, and 19.0%, respectively), the $Q_{y}$ excitation energies increased gradually. Compared with model 1, the $Q_{y}$ excitation energies of model 2 and model 3 were slightly lower, being about 10 and 20 meV, respectively. Among them, the $Q_{y}$ excitation energy of model 3 by TDDFT/ωB97X is in excellent agreement with the experimental one. The three models in gas phase were also computed for comparison, but the $Q_{y}$ excitation energies became a little worse. These results indicate clearly that dispersion correction is necessary to get a reasonable stack structure of THF-Znchl because of the large polarization of the delocalized π bond in the chlorin ring. When the THF solvent molecules are close to Znchl, the latter may deform and generate an induced dipole moment and a corresponding dispersion. Obviously, an implicit solvent model cannot accurately describe this interaction, and therefore the combination of the explicit solvent model of the THF-Znchl dimer and the implicit solvent model by SMD is more suitable for calculating the $Q_{y}$ excitation energy of the Znchl monomer in solution.

**Figure 4:**
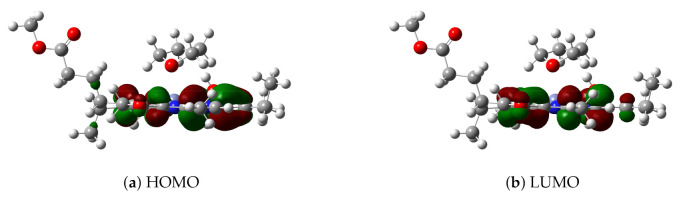
(**a**) HOMO and (**b**) LUMO of model 3.

For the ωB97X/def2-TZVP level, the highest occupied and lowest unoccupied molecular orbitals (HOMO and LUMO, respectively) of model 3 in implicit solvent THF have been plotted in Figure 4. TDDFT results show that the first excited state of model 3 is mainly due to the HOMO→LUMO excitation by 84%; thus, HOMO and LUMO may be used to analyze the excitation mode and the change of electronic density distribution in the first excited state. Since HOMO and LUMO are distributed only in the chlorin ring instead of the THF ligand, this is basically a local excitation. Consequently, both DOOP values and HOMO-LUMO analysis indicate that the THF ligand molecule affects the $Q_{y}$ excitation energy of Znchl by changing its ground state structure, which has also been found in some analogues (for example, see the recent results of chlorophyll-a [55]).

### 3.2. Aggregate

#### 3.2.1. Coupling Strength and QTAIM Properties

**Figure 5:**
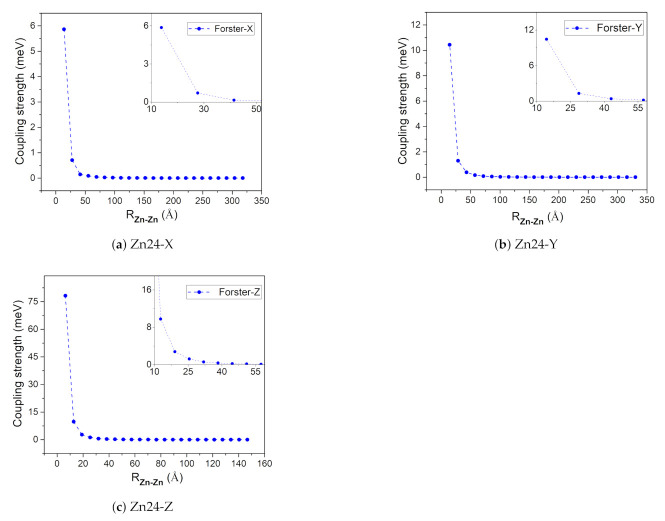
The absolute coupling strength between the first molecule and the remaining 23 molecules in the Zn24-X/Y/Z model.

The coupling strengths of Zn24-X/Y/Z by the Förster coupling theory are plotted in Figure 5 as functions of intermolecular distance (measured by the Zn–Zn distance). With this method, the transition dipole moment in vacuum is 5.0 Debye in the N21→N23 direction, which may be used to estimate the coupling strength through Equation (6) under the point dipole approximation. Figure 5 shows that the coupling strength decreases sharply with the increase of the Zn–Zn distance. The largest coupling strength was obtained for the first two Znchl monomers in the Zn24-Z model (i.e., at the first point in Figure 5c), being −80 meV or −645 cm${}^{-1}$, which is just in the typical range between −550 and −750 cm${}^{-1}$ estimated from chlorosomes [1].

**Figure 6:**
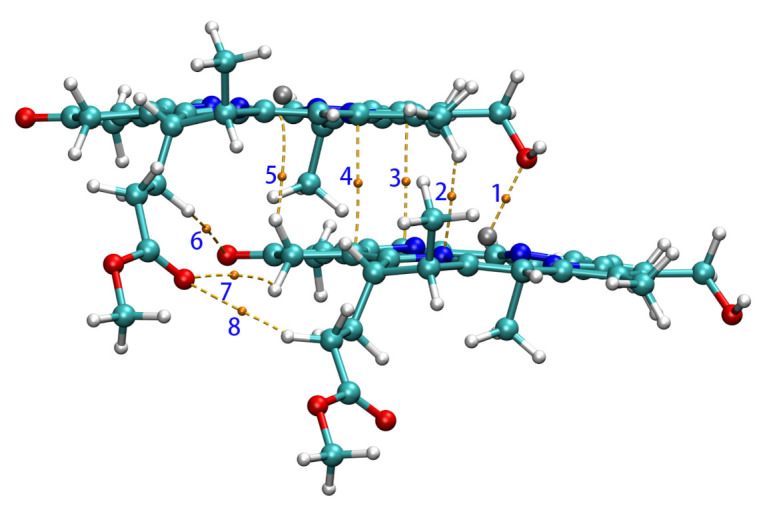
BCPs (in yellow) and paths between two Znchl monomers in the z-direction.

In the QTAIM analysis of Zn-zdimer, eight BCPs in total of type (3,−1) were found between the two fragments (namely, BCP*n*, *n* = 1, 2, ⋯, 8), as seen in Figure 6. BCPs 6, 7, and 8 were located on three weak hydrogen bonds with the O–H distances of 3.121, 3.171, and 3.296 Å, respectively. The QTAIM properties at these BCPs have been collected in Table 3, including critical electron density ($\rho_{c}$), energy density ($H_{c}$), and kinetic energy density ($G_{c}$). BCP1 has a considerable electron density (>0.01 a.u.), since its $H_{c}$ value is negative [56], whereas the ratio $G_{c}/\rho_{c}$ = 1.25 is much larger than 1.0 [57]; BCP1 corresponds to a strongly polarized covalent interaction, or more specifically, a coordination interaction between the Zn and O atoms, this being consistent with experimental findings [15]. The other BCPs have smaller electron densities (<0.01 a.u.) and thus the ratio $G_{c}/\rho_{c}$ is numerically unstable and is not meaningful any more. Their positive $H_{c}$ values indicate that there are more electrostatic (or ionic) interactions than orbital overlap (or covalent) ones. The QTAIM results show that the main interaction between the fragments in Zn-zdimer is the electrostatic one, indicating that the point-dipole approximation is suitable for the aggregates.

**Figure 7:**
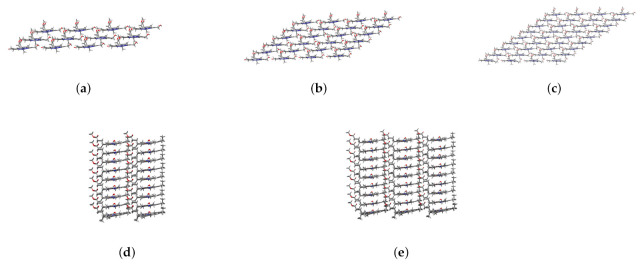
Main views of (**a**) Zn12, (**b**) Zn24, and (**c**) Zn36 and left side views of (**d**) Zn72 and (**e**) Zn108.

When the coupling strength $H_{1j}$ is small enough, the Zn24-X/Y/Z model may be simplified to a smaller one. If $|H_{1j}|\geq$ 0.14 meV is taken as a threshold, jx, jy, and jz determined from Figure 5 are 3, 4, and 9, respectively. Accordingly, four aggregate models, i.e., 4 × 1 × 3 (denoted as Zn12), 4 × 1 × 6 (Zn24), 4 × 1 × 9 (Zn36), 4 × 2 × 9 (Zn72), and 4 × 3 × 9 (Zn108) can be constructed, which may be used to explore the size effect on $Q_{y}$ excitation energies. The structures of these aggregate models are shown in Figure 7.

#### 3.2.2. Spectral Simulation of Aggregates

The absorption spectra of the five different scale aggregations (see Figure 7) were simulated by two methods.

**Figure 8:**
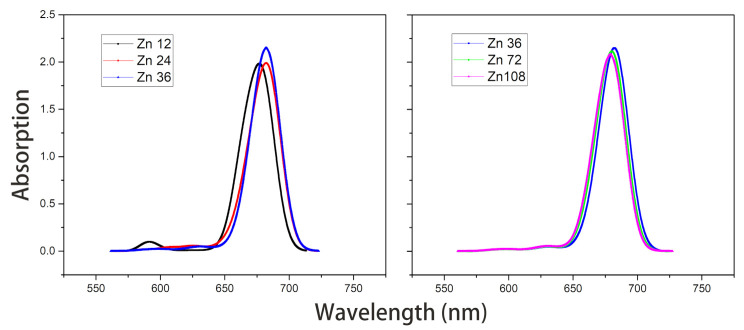
Absorption spectra of model Zn12/24/36 and model Zn36/72/108 in vacuum.

**Figure 9:**
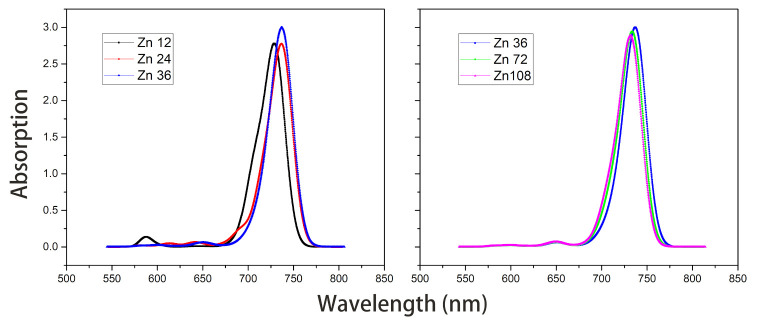
Absorption spectra of model Zn12/24/36 and model Zn36/72/108 with the SMD solvent model.

1. Under the point dipole approximation, the transition dipole moment and site energy of Znchl monomer in a aggregate were calculated in vacuum. From the transition dipole moment of 5.00 Debye, the coupling strength between two molecules in the open dimer was estimated to be about –65 meV.
  The peak positions in the $Q_{y}$ absorption spectra of Zn12, Zn24, and Zn36 (see Figure 8) were 676, 681, and 682 nm, respectively, being gradually red-shifted with the extension of the aggregate in the z-axis direction. However, the absorption spectrum is blue-shifted by an expansion in the x-axis. For example, compared with the absorption peak of the Zn36 model (682 nm), the peaks of Zn72 and Zn108 aggregates were blue-shifted by 2 nm (680 nm) and 4 nm (678 nm), respectively.
  The average site energy and the corresponding wavelength of 36 molecules in the Zn36 aggregate were, respectively, 1.973 eV and 628 nm; the experimental values were 1.917 eV and 647 nm for the Znchl monomer [15]. Thus, the absorption peak of Zn36 is red-shifted by 54 nm in theory relative to the former wavelength; however, it is not consistent with the experimental peak of 92 nm [15].
2. The Zn aggregate was experimentally measured in a 1% (vol/vol) THF–hexane solution [15]. In order to simulate the experimental conditions, the Znchl monomer in the aggregates was calculated with the SMD implicit mixed solvent model. The obtained transition dipole moment was 6.18 Debye, this being significantly larger than the 5.00 Debye in the vacuum, and the coupling strength between two molecules in the open dimer was about –98 meV.
  The peaks in the $Q_{y}$ absorption spectra of Zn12, Zn24, and Zn36 (see Figure 9) were 729, 736, and 737 nm, respectively, so the $Q_{y}$ absorption spectrum of Zn36 tends to a limit value. Similarly to the vacuum case, the extension of the aggregate in the z-axis direction may gradually red-shift the $Q_{y}$ absorption spectrum, and the extension in the x-axis from Zn36 to Zn72 (734 nm) leads to a blue-shift of only 3 nm. Compared with the average absorption peak at 645 nm of 36 molecules in the Zn36 aggregate, the absorption peak of the Zn36 aggregate is red-shifted by 90 nm, being in good agreement with the experimental one of 92 nm [15].

In brief, the $Q_{y}$ absorption spectra of the five aggregates calculated by the two methods exhibit the same trend, i.e., the y and z-direction extensions lead to a red-shift in the absorption spectra of aggregates, whereas the x-direction extension results in a blue-shift. In the exciton theory, the former two and the latter extensions are called J-type aggregates and H-type aggregates [22], respectively.

Among the above two groups of results, the second one is superior to the first one. The excellent agreement with the experimental absorption spectrum indicates the rationality of the layered aggregate constructed. The binding energies of the adjacent dimer in the y and z-directions were further calculated at the ωB97X/def2-TZVP level, and their results were −10.68 Kcal/mol and −25.31 Kcal/mol, respectively. Therefore, we believe that the hydrogen bond in the $3^{1}$–13${}^{1}$ position in the y-direction adjacent dimer and the coordination bond between $3^{1}$ O and Zn in Zn-zdimer are conducive to the formation of J aggregates, which can extend the absorption spectrum of the monomer to the infrared region. The results also indicates that the z-direction is the dominant aggregation direction of the aggregate, which suggests that the accumulation in z-direction should be mainly controlled in the experimental synthesis of Znchl aggregates.

## 4. Conclusions

The $Q_{y}$ excitation energies of the Znchl monomer model and Znchl supramolecular aggregates were simulated by TDDFT and exciton theory respectively. Using the ωB97X functional combined with the SMD solvent model, the $Q_{y}$ excitation energy by TDDFT was in good agreement with the experimental excitation energy, which confirms that the THF molecule forms axial coordination with the Zn atom in Znchl.

The results of the parallel-stacked, stepped, layered aggregates show that the essential factors affecting the $Q_{y}$ excitation energy of the aggregates are the intermolecular coupling strength and the site energy, and therefore the surrounding solvents have to be taken into account. Using the coupling strength between molecules by Förster’s theory with a distance-dependent solvent shielding factor, the experimental $Q_{y}$ absorption spectrum has been well reproduced, meaning that the structures of layered aggregates are reasonable. The binding energy results show that the z-direction is the dominant aggregation direction of Znchl aggregates, and stacking in this direction can effectively expand the monomer absorption spectrum into the near infrared region; this is good information for the synthesis of light-harvesting antennas. In addition, the obtained structure model and Hamiltonian at the atomic level provide a necessary basis for the further simulation of ultra-fast spectrum and energy transfer in the artificial light-harvesting antenna, and are inspirational for the development of artificial light capture antennas in experiments.

## Figures and Tables

**Table 1:** Key bond lengths (in Å) and bond angles (in degrees) of model 1, model 2, and model 3.

Structural Parameter	Model 1	Model 2	Model 3
Zn-N21 Zn-N22 Zn-N23 Zn-N24 $C13^{1}=O$ $C17^{3}=O$	1.979 2.050 1.972 2.190 1.219 1.212	2.016 2.080 2.011 2.224 1.220 1.212	2.014 2.074 1.998 2.193 1.219 1.212
N21-Zn-N22 N22-Zn-N23 N23-Zn-N24 N24-Zn-N21 N21-Zn-N23	92.4 88.8 88.6 90.2 178.1	90.5 87.2 86.9 88.8 158.3	90.8 87.2 88.3 88.9 160.8
DOOP	0.03	0.38	0.33

**Table 2:** $Q_{y}$ excitation energies and oscillator strengths of the three models.

Functional	Model 1		Model 2		Model 3	
	$Q_{y}$/eV ( λ/nm)	f	$Q_{y}$/eV (λ/nm)	f	$Q_{y}$/eV (λ/nm)	f
	in THF solvent					
LC-ωPBE ωB97X CAM-B3LYP	1.902 (652) 1.938 (640) 2.038 (608)	0.28 0.29 0.32	1.892 (655) 1.927 (643) 2.027 (612)	0.25 0.26 0.30	1.879 (660) 1.915 (648) 2.018 (614)	0.26 0.27 0.30
	in vacuum					
LC-ωPBE ωB97X CAM-B3LYP	1.935 (641) 1.977 (627) 2.089 (594)	0.19 0.19 0.21	1.924 (644) 1.965 (631) 2.076 (597)	0.17 0.18 0.19	1.909 (650) 1.951 (635) 2.067 (600)	0.17 0.18 0.19
Expt. [14]	1.917 eV (647 nm)					

**Table 3:** QTAIM properties of Zn-zdimer at bond critical points (BCPs) (in a.u.).

Property	BCP1	BCP2	BCP3	BCP4	BCP5	BCP6	BCP7	BCP8
	$O_{81}$ ${-\mathrm{Zn}}_{1}$	$H_{125}$ ${-N}_{9}$	$C_{112}$ ${-C}_{12}$	$C_{86}$ ${-C}_{16}$	$C_{96}$ ${-H}_{61}$	$H_{140}$ ${-O}_{2}$	$O_{79}$ ${-H}_{62}$	$O_{79}$ ${-H}_{66}$
$\rho_{c}$ $H_{c}$ $G_{c}$	0.0458 −0.0021 0.0572	0.0082 0.0012 0.0055	0.0051 0.0007 0.0030	0.0046 0.0007 0.0028	0.0072 0.0007 0.0043	0.0028 0.0006 0.0019	0.0023 0.0006 0.0016	0.0014 0.0004 0.0010

## Data Availability

Data is contained within the submitted article.

## Abbreviations

The following abbreviations are used in this manuscript:

Bchl	bacteriochlorophyll
BCP	bond critical point
DOOP	out-of-plane distance
HOMO	the highest occupied molecular orbitals
LUMO	the lowest unoccupied molecular orbitals
QTAIM	quantum theory of atoms in molecules
SMD	implicit solvation model based on density
TDDFT	time-dependent density functional theory
Zn-zdimer	Zn-centered chlorin dimer stacked in z-direction
Znchl	Zn-centered chlorin
